# Orchestration of hepatocyte inflammatory responses by monocytes during acute viral hepatitis in ducks in vitro

**DOI:** 10.1186/s13567-025-01630-9

**Published:** 2025-10-17

**Authors:** Lizhen Gong, Xiaoming Lin, Yajia Gou, Yi Liu, Jingwen Dong, Di Sun, Sai Mao, Shun Chen, Mafeng Liu, Dekang Zhu, Mingshu Wang, Renyong Jia, Shaqiu Zhang, Ying Wu, Xinxin Zhao, Juan Huang, Qiao Yang, Bing Tian, Zheng Wu, Yu He, Anchun Cheng, Xumin Ou

**Affiliations:** 1 Engineering Research Center of Southwest Animal Disease Prevention and Control Technology, Ministry of Education of the People’s Republic of China, Sichuan Agricultural University, Chengdu, Sichuan China; 2 Agricultural Animal Diseases and Veterinary Public Health Key Laboratory of Sichuan Province, Chengdu, Sichuan China; 3 Institute of Veterinary Immunology and Green Drugs, Veterinary Department in College of Aminal Science, State Key Laboratory of Green Pesticide, Guizhou University, Guiyang, 550025 China; 4 Institute of Veterinary Medicine and Immunology, College of Veterinary Medicine, Sichuan Agricultural University, Chengdu, China

**Keywords:** Viral hepatitis, tRNAome, PBMCs, Monocyte, DHAV

## Abstract

Severe liver inflammation, in which the interaction between circulating immune cells and hepatocytes plays a crucial role, is a major concern in acute viral hepatitis. Nevertheless, the mechanisms by which these circulating immune cells drive hepatocyte inflammatory responses remain inadequately understood. In this study, we demonstrate that the transcription of several notable proinflammatory cytokines, including IL-1β, IL-6, and TNF-α, is markedly greater in cocultures of duck hepatitis A virus (DHAV)-infected hepatocytes than in hepatocytes infected with only DHAV. Simultaneously, the remodelling of the mature tRNAome in infected hepatocytes is orchestrated by coculturing peripheral blood mononuclear cells (PBMCs). Consistent with the effects of PBMC coculture, monocyte coculture also enhances the transcription of proinflammatory cytokines and the remodelling of the mature tRNAome in infected hepatocytes. These results indicate the importance of monocytes in orchestrating proinflammatory cytokine transcription and mature tRNAome remodelling in infected hepatocytes. Importantly, the coculture of monocytes with DHAV-infected hepatocytes not only regulates IL-1β transcription and mature tRNAome remodelling but also orchestrates protein translation within infected hepatocytes. Furthermore, neutralizing secreted IL-1β proteins influences the transcription of downstream cytokines associated with IL-1β signalling. Collectively, our findings reveal that monocytes play a crucial role in orchestrating the inflammatory response of hepatocytes through mechanisms involving the transcriptional activation of proinflammatory cytokines and the coordination of tRNAome remodelling-mediated translation.

**Supplementary Information:**

The online version contains supplementary material available at 10.1186/s13567-025-01630-9.

## Introduction

Viral hepatitis is a type of liver inflammatory disease [1]. The extent of liver inflammation, which can manifest as acute or chronic viral hepatitis, is influenced by the specific virus involved and the proinflammatory response of the host [2]. Previous studies reported that ducklings infected with duck hepatitis A virus (DHAV) exhibit acute viral hepatitis characterized by apparent hepatocyte damage and immune cell infiltration [3, 4]. The infiltrating immune cells may include Kupffer cells (i.e., liver macrophages), T cells, dendritic cells and natural killer T cells, all of which originate from the circulatory system [5]. The recruitment of these immune cells to the liver is supposed to eradicate the viral infection; however, it may also lead to adverse immunopathological effects, such as overreactive inflammation [6]. In a mouse model, increased numbers of virus-specific T cells within liver tissue can exacerbate hepatocyte damage [7, 8]. Additionally, human monocytes, which serve as progenitor cells for liver Kupffer cells, have also been implicated in the development of viral hepatitis [9]. These findings from human and veterinary studies strongly suggest that these infiltrating immune cells vary functionally in orchestrating liver inflammation. Although the role of infiltrating immune cells in duck viral hepatitis was previously implicated in the acute form of duck viral hepatitis caused by DHAV infection [3, 10, 11], the underlying proinflammatory mechanisms remain inadequately understood.

In acute duck viral hepatitis, there is significant and often excessive activation of the proinflammatory cytokine response in liver tissues, as we previously reported [3, 12]. The proinflammatory cytokines involved include IL-1, IL-6, and TNF-α. Similarly, in humans, an increase in these cytokines can lead to systemic inflammation, which is observed in viral hepatitis patients, as well as in those populations infected with coronaviruses and avian influenza viruses [13, 14]. Emerging investigations indicate that these proinflammatory cytokines are produced not only by tissue-resident immune cells, such as monocytes [15] but also by other cells with which they interact, such as hepatocytes [16, 17]. For example, blocking IL-1 receptors in hepatocytes with an antagonist can inhibit IL-1β-induced inflammation, thus lowering liver inflammation [17], suggesting the importance of hepatocytes in liver inflammation. During acute viral hepatitis, these infected hepatocytes are closely colocalized with circulating immune cells, as determined by histopathological analysis [12]. However, the effects of circulating immune cells on hepatocyte inflammatory responses remain poorly understood.

When proinflammatory cytokines are transcribed from DNA, a crucial step for these transcripts to become functional is the translation of their mRNAs into proteins [18]. Theoretically, the expression of proinflammatory cytokine proteins requires coordination with the mature host tRNAome that decodes them [15, 19]. The decoding function remains central to the mature tRNAome. tRNA facilitates the incorporation of amino acids into the growing polypeptide chain by decoding mRNA codons sequentially [20, 21]. However, the levels of the tRNAome can vary significantly under different physiological and pathological conditions [15]. We previously developed a method to quantify the tRNAome and study changes in the mature human tRNAome [22]. Indeed, we found that the mature tRNAome significantly varies in the context of liver cancer and viral infections [15, 23]. For example, we recently showed that the mature tRNAome in human macrophages was altered following infection with the hepatitis E virus [15]. In other settings, evidence has demonstrated that the abundance of many tRNAs in COVID-19 patients with severe cytokine storms [24] is significantly altered compared with that in healthy individuals [25], and a similar observation was made in the mould species *Aspergillus niger* infected with adenovirus type-6 (AdV-6) [26]. These observations strongly show the commonness of tRNAome remodelling among organisms infected with different pathogens, but its biological relevance in viral infections is only partially understood.

In this study, we aimed to elucidate the influence of circulating lymphocytes on hepatocyte inflammatory responses in the context of duck acute viral hepatitis. We employed a coculture system of peripheral blood mononuclear cells (PBMCs) and hepatocytes to mimic natural DHAV infection in vitro. Our findings indicated that circulating monocytes predominantly promoted the transcription of proinflammatory cytokines in hepatocytes, concurrently facilitating the remodelling of the mature tRNAome. The role of monocytes in orchestrating the transcription of IL-1β, remodelling of the mature tRNAome and protein synthesis was revealed. This study revealed a previously unrecognized function of monocytes in modulating the inflammatory response of hepatocytes by coordinating the transcription of proinflammatory cytokines and tRNAome remodelling-mediated protein translation.

## Materials and methods

### Virus and animals

DHAV (GenBank No: JQ301467.1) was obtained from the Engineering Research Center of Southwest Animal Disease Prevention and Control Technology at Sichuan Agricultural University. The viral stock utilized in this study was derived from the allantoic fluid of 9–11-day-old DHAV-infected duck embryos. The virus titre was quantified at 1.0 × 10^9^ copies per mL, as measured by a qPCR method [27]. Sixteen-day-old duck embryos from Ya'an Farm of Sichuan Agricultural University were consistently used for hepatocyte preparation, while 11-day-old embryos were used for fibroblast preparation, and 26-day-old embryos were used for enterocyte preparation. In addition, two mature Peking ducks were raised in separate DHAV-free environments. At four months of age, blood samples from the two ducks were used to isolate PBMCs for subsequent coculture experiments. At the conclusion of this project, the ducks were six months old.

### Culturing of duck primary fibroblasts, hepatocytes and enterocytes

Duck primary fibroblasts were prepared from 11-day embryos following a previously established approach [28]. Owing to the limited mass of liver or intestinal tissue obtained from smaller embryos, 16-day-old and 26-day-old embryos were selected for the isolation of hepatocytes and enterocytes, respectively. A combination of mechanical dissociation and collagenase digestion was used for duck primary hepatocytes (DPHs) [29]. Briefly, the liver was immersed in ice-chilled phosphate-buffered saline (PBS). Once the PBS became clear, indicating the absence of visible red blood cells, the liver tissue was sectioned into small fragments approximately 2–3 mm wide using a sterile surgical blade. These tissue pieces were transferred to a 10 mL tube and rinsed twice with PBS. Subsequently, 1 mL of 0.1% type II collagenase (Solarbio, P8150) was added for digestion at 37 °C, with gentle shaking every 5 min. Following digestion, 4 mL of Dulbecco's modified Eagle’s medium (DMEM) was added to halt the enzymatic activity, and pipetting tips were used to disperse the hepatocytes from the remaining liver tissue. The resulting cell dissociation solution was placed on ice for two minutes to facilitate the aggregation of larger tissue fragments. The supernatant was filtered through a 70 μm cell strainer and centrifuged at 2000 rpm for five minutes at 4 °C. The resulting hepatocyte pellet was resuspended in DMEM (BasalMedia, P110L7) supplemented with 10% foetal bovine serum (FBS) (NEWZERUM, CP500) and 1% penicillin‒streptomycin (Solarbio, P1400). Once the hepatocytes had adhered to the culture plate, the medium was changed for subsequent coculture assays.

For duck primary enterocytes, the entire intestine from 26-day-old duck embryos was extracted and immersed in ice-chilled PBS, followed by a vertical incision via a small syringe needle. The intestine was subsequently cut into ~3–5 mm3 pieces with scissors. The intestinal tissues were digested with 2 mL of type II collagenase (Solarbio, P8150) at 37 °C for 50 min. The digested tissue pieces were thoroughly washed to disassociate the enterocytes. The resulting cell suspension was filtered through a 100 μm cell strainer and centrifuged at 3500 rpm and 4 °C for 5 min. The cell pellets were resuspended and cultured in the same medium used for hepatocyte culture.

### Oil red O staining and albumin measurement

Hepatocytes are characterized by a high lipid content and the ability to secrete albumin. To validate these characteristics in cultured DPHs, we utilized duck primary fibroblasts and human hepatocytes as negative and positive controls, respectively. Lipid droplet and albumin secretion were assessed according to the manufacturer’s protocols via an Oil Red O staining kit (Jiancheng Bioengineering, D027) and an albumin assay kit (Jiancheng Bioengineering, A028).

### Immunofluorescence assay

Slides of DPHs, duck fibroblasts, and human hepatocytes (Huh 7.0 cell line) were harvested by fixation with 4% paraformaldehyde, permeabilized with 0.3% Triton X-100, and blocked with 5% BSA. A rabbit anti-human HNF4α (a notable hepatocyte marker) antibody (Thermo Fisher, PA5-26443) was used as the primary antibody (1:1000), as it targets an identical peptide between humans (UniProt: P41235, amino acids 107‒139) and ducks. Duck monocytes were separated and cultured on slides [30]. Then, the monocytes were stained and identified with a rabbit anti-CD68 polyclonal antibody (Boster Biological Technology, A006021, 1:400) according to previous publications [31]. The secondary antibody used was Alexa Fluor 488-labelled goat anti-rabbit IgG (1:1000) (Yeasen, China). Nuclei were stained with DAPI (1:200) (Solarbio, C0065), and the slides were subsequently covered with glycerol and examined via a fluorescence microscope (BX53, OLYMPUS).

### PBMC and monocyte isolation

Blood samples (4 mL) were collected from the duck jugular vein for the isolation of PBMCs via a lymphocyte separation reagent kit (Solarbio, P5720) following the manufacturer’s instructions. Monocytes were further separated from fresh PBMCs by differential adhesion as previously described [30] and were validated by a rabbit anti-CD68 polyclonal antibody (Boster Biological Technology) [31]. The isolated PBMCs and monocytes were resuspended in RPMI 1640 medium (BasalMedia, P210LV) supplemented with 10% FBS and 1% penicillin‒streptomycin and diluted to a concentration of 5.0 × 10^6^ cells/mL. All procedures for PBMC isolation were conducted expeditiously, and the cells were used within 2 h. The density of the isolated PBMCs was determined via a hemocytometer, and the specified number of PBMCs was used in subsequent experiments.

### Coculture of PBMCs and hepatocytes

DPHs were seeded at a density of 1.0 × 10^5^ cells/well in 12-well plates, and three experimental conditions were evaluated and compared: (i) pure culture of the DPHs, (ii) DPHs infected with DHAV (2.0 × 10^7^ copies per well), and (iii) coculture of the DHAV-infected DPHs with PBMCs (5.0 × 10^6^ per well). Following a 24-h culture, the DPHs were (or not, condition i) inoculated with DHAV for 2 h (condition ii), followed by 24 h of PBMC coculture (condition iii). The PBMCs in the medium were removed by washing the wells 2‒3 times with 1‒2 mL of phosphate-buffered saline (PBS). All samples were collected at a time point equal to condition iii. After confirming the absence of visible PBMCs via a microscope, the hepatocytes were treated with RNAiso Plus reagent (Perfect Real Time, Takara, Cat# 9108) for RNA isolation.

### RT‒qPCR

Total RNA was extracted via RNAiso plus and measured via a NanoDrop (Thermo Scientific, Wilmington, DE). cDNA synthesis was subsequently performed with PrimeScript™ RT Master Mix (Takara, Cat# RR036A) according to the manufacturer’s instructions. The transcription levels of proinflammatory cytokines were quantified via Taq Pro Universal SYBR qPCR Master Mix (Vazyme, Q712) with a real-time PCR system (Bio-Red CFX96). The cycling conditions were 95 °C for 3 min, 45 cycles of 95 °C for 30 s, 58 °C for 10 s, and 72 °C for 30 s, followed by the melt curve stage of 95 °C for 15 s, 60 °C for 1 min and a 0.7 °C stepwise increase until 95 °C was reached. The primers used are listed in Additional file 1.

### Quantification of the mature tRNAome

Previously, we developed a simple qPCR approach to quantify the mature tRNAome in the setting of human cells [22]. Using this established approach, we redesigned primer sequences to specifically target the mature tRNAome in ducks (Additional file 2). This approach uses a universal DNA/RNA hybrid tRNA adaptor that can precisely connect to the 3’CCA terminal of all mature tRNA species. For details of mature tRNAome quantification, the step-by-step protocol can be found in our previous publication [22].

### Proteomic analysis of tRNA biology-related proteins in the liver

The protein data associated with tRNA biology were obtained from an ongoing project investigating the effects of DHAV infection on the liver proteome. The raw proteomic data have been deposited at the Figshare platform and are publicly available [32]. Duckling liver samples, both infected and uninfected with DHAV, were analysed 24 hours post-infection (hpi) via tandem mass tag (TMT)-based proteomic quantification. Three biological replicates were conducted for both the infected and control groups.

### Analysis of tRNA fragmentation

Duck primary fibroblasts infected with DHAV were used for small RNA sequencing [33]. Small RNA sequencing and analysis were performed by OE Biotech Co., Ltd. (Shanghai, China). The initial reads were converted into sequence data (commonly referred to as raw data) through base calling, followed by filtration of low-quality reads. Reads shorter than 15 nucleotides and longer than 41 nucleotides were excluded, resulting in the acquisition of clean reads. Noncoding RNAs are categorized as ribosomal RNAs (rRNAs), transfer RNAs (tRNAs), small nuclear RNAs (snRNAs), and small nucleolar RNAs (snoRNAs). The tRNA-derived fragments were subsequently compared between the infected and uninfected cells.

### Indirect coculturing of PBMCs and hepatocytes

DPHs were seeded at a density of 1.0 × 10^5^ cells/well in 12-well plates under four different experimental conditions: (i) DPHs; (ii) DPHs infected with DHAV (2.0 × 10^7^ copies/well); (iii) cocultures of DHAV-infected DPHs with PBMCs at a concentration of 5.0 × 10^6^ cells/well; and (iv) cocultures of DHAV-infected DPHs with an equivalent number of transwell-separated PBMCs (5.0 × 10^6^ cells/well) using a membrane with a pore size of 3 μm (LABSELECT, 14,211). After 24 h of coculture, the PBMCs were either washed away under Condition III or removed when the inside chamber was removed under Condition IV. RNA samples from hepatocytes were subsequently collected for cytokine and tRNAome quantification.

### Western blotting

The cells were washed with PBS, lysed with RIPA buffer, and centrifuged to yield a clear supernatant. The lysate was transferred to a new tube, combined with 6 × protein loading buffer (TransGen Biotech, DL101) and boiled in a water bath for 10 min. The samples were subjected to 12% SDS‒PAGE. The proteins were transferred to a PVDF membrane. The primary antibodies used included a rabbit anti-duck IL-1β polyclonal antibody (1:1000) (a gift from Dr Sai Mao) and a mouse anti-duck β-actin antibody (1:5000) (TransGen Biotech, HC201). The secondary antibodies used included HRP-labelled goat anti-rabbit IgG (1:3000) and HRP-labelled goat anti-mouse IgG (1:3000). Enhanced chemiluminescence (ECL) images were captured using a ChemiDoc MP Imaging System (Bio-Rad, USA).

### IL-1β protein neutralization assay

The supernatant of the IL-1β protein mixture was neutralized with a rabbit anti-IL-1β polyclonal antibody, as described for the western blots. In the present study, 5.0 × 10^6^ cells, 2 mg/mL and 4 mg/mL IL-1β antibody were added to determine the presence of a dose-dependent effect [34]. The efficacy of IL-1β protein neutralization at the two indicated doses was assessed by quantifying downstream cytokines associated with IL-1β signalling.

### Statistical analysis

Relative gene expression was calculated via the 2^−ΔΔ^C_T_ method, with glyceraldehyde 3-phosphate dehydrogenase (GAPDH) used as a reference gene for normalization of target gene expression. All the data are presented as the means ± standard deviations (SDs). Statistical significance was determined via unpaired t tests, one-way ANOVA, or two-way ANOVA with Dunnett’s multiple comparisons, with a *p* value of less than 0.05 considered statistically significant.

## Results

### DPHs are suitable for treating DHAV infection but do not elicit robust inflammatory cytokine responses

**Figure 1:**
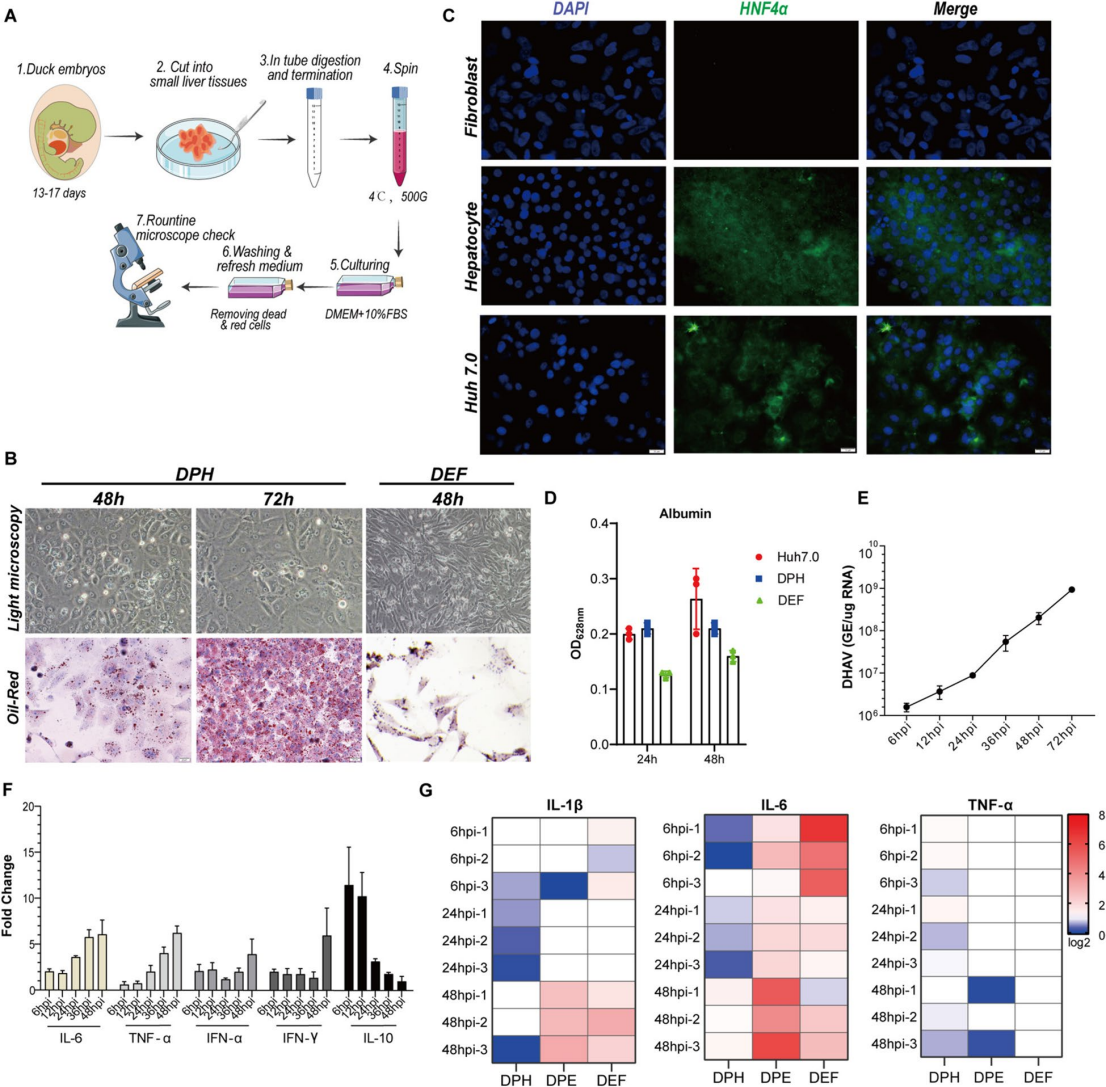
**Culturing of duck primary hepatocytes and their suitability for DHAV infection**.** A** Preparation of duck primary hepatocytes (DPHs). For detailed procedures, see Materials and methods. **B** Light microscopy images and Oil Red O staining of cultured DPHs and ducked embryonic fibroblasts (DEFs). **C** Immunofluorescence staining of the expression of hepatocyte nuclear factor 4 alpha (HNF4α) in duck fibroblasts and hepatocytes through an immunofluorescence assay. Human Huh7.0 cells were used as a positive control. **D** The secretion of albumin was measured in DPHs and Huh7.0 cells. The duck fibroblasts were utilized as the control group. **E** Replication curve for DHAV in cultured DPHs. The quantification of DHAV RNA was performed via RT‒qPCR. Three biological replicates were utilized, with each replicate comprising a distinct well number that seeded the same number of hepatocytes derived from the same embryo (*n* = 3). The data are presented as the means ± standard deviations (SDs). **F** The expression of cytokines, including IL-6, TNF-α, IFN-α, IFN-γ, and IL-10, in DPHs induced by DHAV infection was quantified via RT‒qPCR. Three biological replicates were utilized, with the time points being the same as those in Panel E. Data are presented as the means ± SDs. **G** A comparative analysis of DHAV-induced proinflammatory cytokines in DPHs, DEFs, and duck primary enterocytes (DPEs) was conducted. The RT‒qPCR quantification data were used to create heatmaps.

Following the procedure for culturing human hepatocytes, a pipeline for culturing DPHs was established [29]. Briefly, the livers of 16-day-old duck embryos were dissected and subjected to digestion with collagenase II (Figure 1A). The resulting dissociated DPHs were centrifuged, resuspended, and cultured in DMEM supplemented with 10% FBS. After 24 h of culture, the cells formed a uniform cell monolayer and exhibited a typical hepatocyte morphology, with an apparent nucleus and nucleoli visible under a light microscope. Oil Red O staining revealed numerous lipid droplets within the cytoplasm of the DPHs, a characteristic feature of hepatocytes. However, this staining was minimal in duck embryonic fibroblasts (DEFs) (Figure 1B). Immunofluorescence assays revealed high expression of the hepatocyte-specific marker hepatocyte nuclear factor 4 alpha (HNF4α) in both human and duck hepatocytes but not in DEFs [35] (Figure 1C, bottom and top panels, respectively). The functionality of the DPHs was also confirmed through an albumin secretion assay (Figure 1D). However, prolonged culture of the DPHs for more than 96 h resulted in a fibroblast-like cell morphology (Additional file 3), which is similar to that observed in mouse and human primary hepatocyte cultures [36].

Next, we assessed the suitability of DPHs for infection with DHAV. The results indicated that DPHs infected with a low dose (1.0 × 10^6^ copies/well) of DHAV exhibited dramatic viral replication, increasing over 1000-fold to approximately 1.0 × 10^9^ copies/μg RNA (Figure 1E). These findings support the appropriateness of DPHs for DHAV infection assays and are consistent with the characterization of DPHs as the target cell type for DHAV. Previous studies demonstrated that ducklings infected with DHAV elicited a robust cytokine response accompanied by significant hepatocyte pathology in liver tissue [3]. In contrast, DPHs infected solely with DHAV exhibited a mild cytokine response (Figure 1F). Furthermore, we compared the proinflammatory cytokine responses of duck embryo fibroblasts (DEFs), DPHs, and duck primary enterocytes (DPEs). Our analysis revealed that DHAV infection in DPHs resulted in a considerably lower cytokine response (Figure 1G). These findings suggest that the inflammatory response in hepatocytes induced by DHAV is relatively minor compared with that observed in infected ducklings, potentially attributable to the absence of immune cell infiltration [3].

### DPHs cocultured with PBMCs increase the transcription of proinflammatory cytokines in DHAV-infected hepatocytes

**Figure 2:**
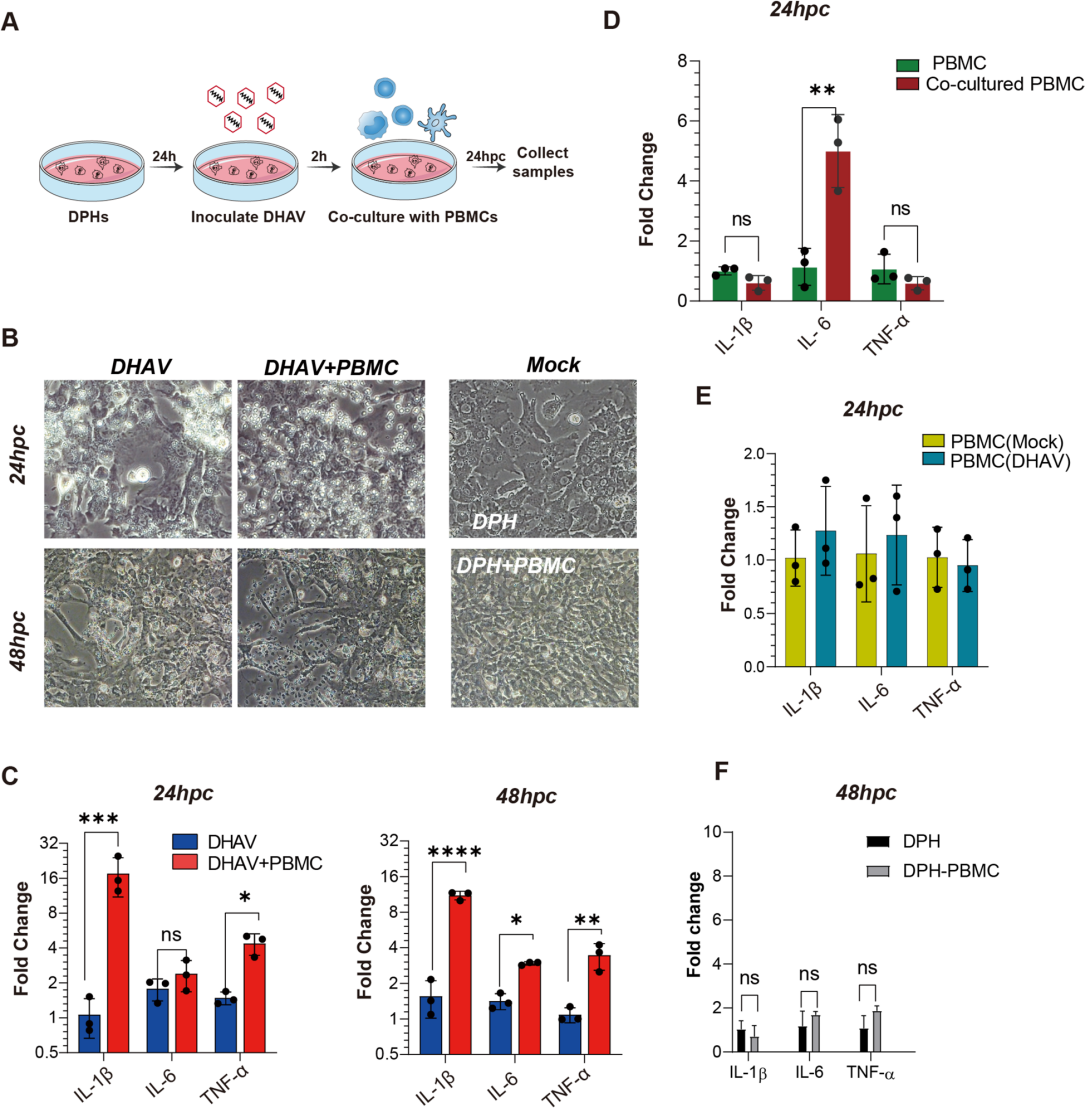
**Coculturing PBMCs amplifies the transcriptional inflammatory response within DHAV-infected hepatocytes**.** A** Following 24 h of preculture, primary hepatocytes were inoculated with DHAV (2.0 × 10^7^ copies per well) for 2 h, after which 24 hpc or 48 hpc of PBMC coculture (5.0 × 10^6^ cells/well) was initiated. The suspended PBMCs were removed through washing with PBS, and total RNA from the cocultured DPHs (panel C) and the cocultured PBMCs (panel D) was then collected for cytokine quantification. **B** Light microscopy images of DHAV-infected DPHs cocultured with or without PBMCs at 24 hpc and 48 hpc. Uninfected DPHs cocultured with PBMCs were used as a negative control (DPH + PBMC), and uninfected DPHs were utilized as a blank control (Mock). **C** DPHs from infected cocultures: The impact of PBMCs on the transcription of proinflammatory cytokines in DHAV-infected DPHs. The PBMCs in the medium were removed by washing with PBS. The levels of cytokines, including IL-1β, IL-6, and TNF-α, in the cocultured DPHs at 24 hpc and 48 hpc were quantified via reverse transcription quantitative polymerase chain reaction (RT‒qPCR). Uninfected DPHs served as a control for normalization of gene expression. Three biological replicates were utilized with each well containing 1.0 × 10^5^ DPHs/well and then cocultured with 5.0 × 10⁶ PBMCs/well (*n* = 3). The data are presented as the means ± SDs. Statistical significance was determined via unpaired t tests; *, *p* < 0.05; **, *p* < 0.01. **D** PBMCs from infected cocultures: Quantification of IL-1β, IL-6 and TNF-α in cocultured PBMCs from the panel C assay. Normal PBMCs were used as control groups. **E** PBMC infection alone: The impact of DHAV infection on the expression levels of cytokines, such as IL-1β, IL-6, and TNF-α, in PBMCs, as quantified via RT‒qPCR. A total of 2.0 × 10⁷ copies per well of DHAV and 5.0 × 10⁶ cells/well of PBMCs were used. Three biological replicates were used (*n* = 3), and the data are presented as the means ± SDs. **F** DPHs from cocultures without DHAV infection: The effects of PBMCs (5.0 × 10⁶ cells/well) on the transcription of proinflammatory cytokines in DPHs were compared and normalized to those of DHAV-uninfected DPHs (set as 1.0).

To date, in vitro inflammation studies have focused predominantly on the infection of either fibroblasts or hepatocytes [37]. However, these experimental conditions do not accurately reflect the actual situation in DHAV-infected livers, where the involvement of immune cells is neglected. Pathology of the livers of DHAV-infected ducks has revealed significant lymphocyte infiltration [4, 12], and these lymphocytes are derived primarily from circulating peripheral blood mononuclear cells (PBMCs). To investigate whether PBMCs contribute to the hepatocyte inflammatory response, we conducted a coculture experiment involving DHAV-infected hepatocytes and PBMCs (Figure 2A). When cocultured with PBMCs, DPHs presented significantly more pronounced cytopathic effects (CPEs) than DPHs infected solely with DHAV did and became particularly evident at 48 hours post-coculture (hpc) (Figure 2B). These findings support the hypothesis that PBMCs play a pivotal role in the pathogenesis of hepatocyte pathology. Furthermore, PBMCs significantly increased the transcription levels of several key proinflammatory cytokines, including IL-1β, IL-6, and TNF-α, to varying extents in infected DPHs (Figure 2C). In this experiment, the supernatant containing PBMCs was removed through washing with PBS; however, deleting RNA from the monocyte populations was challenging because of the plate adherence activity of the monocytes [38]. Our preliminary assessment indicated that RNA contamination from cocultured monocytes constituted only 7–11% of the total hepatocyte RNA (Additional file 4) and was thus unlikely to contribute to the major upregulation of proinflammatory cytokines. Furthermore, in cocultured PBMCs (Figure 2D) or PBMCs solely infected with DHAV (Figure 2E), there was no significant upregulation of the transcription of IL-1β and TNF-α. However, IL-6 was significantly upregulated in the cocultured PBMCs (Figure 2D). Additionally, in uninfected DPHs from cocultures with PBMCs, there was no significant increase in the proinflammatory cytokines IL-1β, IL-6 and TNF-α (Figure 2F). Collectively, these findings suggest that DHAV-infected hepatocytes from cocultures themselves predominantly contribute to proinflammatory cytokine transcription.

To further investigate the conditions under which these PBMCs can increase the transcription of proinflammatory cytokines in DHAV-infected hepatocytes, different numbers of PBMCs, infection doses of DHAV, and coculture times were investigated. Our results indicated that coculturing a high number of PBMCs (5.0 × 10^6^ cells/well) with DPHs (1.0 × 10^5^ cells/well) infected with a suitable DHAV infection titre (2.0 × 10^7^ copies/well) resulted in the most pronounced transcription of proinflammatory cytokines (Additional file 5). Among the various time points assessed, the peak effect was observed at 24 hpc (Additional file 5). In summary, PBMCs significantly increased the transcription of proinflammatory cytokines in infected hepatocytes, with the extent of this effect depending on the number of PBMCs (5.0 × 10^6^ cells/well), the viral titre (2.0 × 10^7^ copies/well), and the duration of coculture (24 hpc). Accordingly, these specific conditions were selected for later investigations.

### PBMC coculturing remodels the mature tRNAome in infected hepatocytes

**Figure 3:**
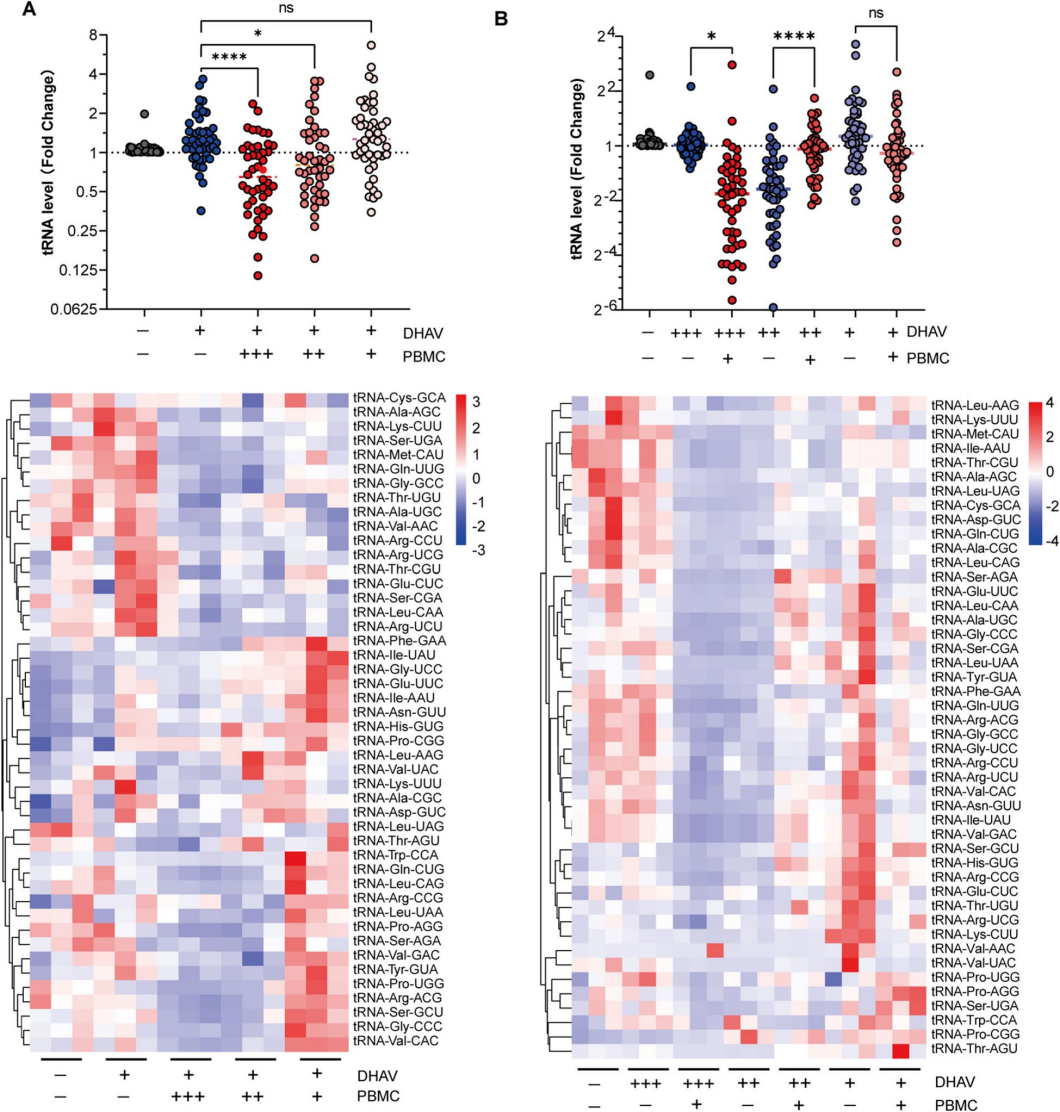
**Coculturing PBMCs induces remodelling of the mature tRNAome in DHAV-infected hepatocytes**.** A** The influence of varying quantities of PBMCs on mature tRNAome remodelling. Scatter plots and heatmaps are displayed. The symbols “+”, “++”, and “+++” represent the quantities of PBMCs used, which are 5.0 × 10⁶, 5.0 × 105, and 5.0 × 104 PBMCs per well, respectively. The “+” symbol to the text "DHAV" signifies 2.0 × 10⁷ copies per well of DHAV. The symbol "-" indicates the absence of either DHAV or PBMCs. Three biological replicates were employed (*n* = 3). Statistical significance was determined via unpaired t tests; *, *p* < 0.05; **, *p* < 0.01; ***, *p* < 0.001. **B** The impact of the DHAV titre on mature tRNAome remodelling. The symbols “+”, “++”, and “+++” represent 2.0 × 10⁷, 2.0 × 10⁶ and 2.0 × 105 copies per well of DHAV being used, respectively. The case of “+” in reference to the text “PBMC” signifies 5.0 × 10^6^ PBMCs per well. The symbol “-” denotes the absence of DHAV or PBMCs. The tRNAome data were normalized to those of the uninfected group (set as 1.0).

**Figure 4:**
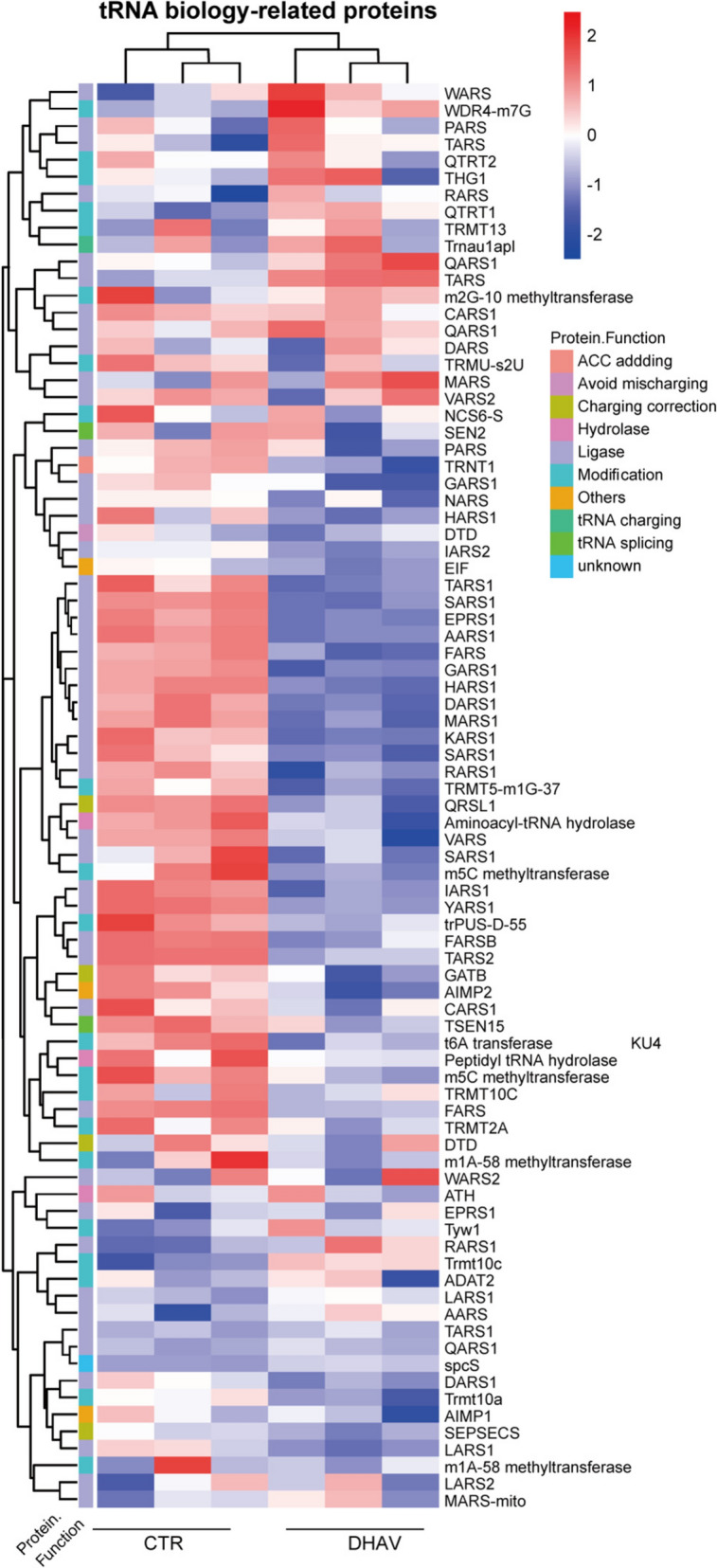
**Heatmap of tRNA biology-related proteomic changes in the livers of DHAV-infected ducklings.** The figure depicts a heatmap of tRNA biological process-related proteins in the livers of DHAV-infected ducklings. The liver samples were collected after 24 h of infection. Liver samples from three individuals infected with DHAV were used for TMT-based proteomic quantification, as were uninfected liver samples (*n* = 3). For more details, see the Materials and methods section.

The mature tRNAome performs translational decoding. Next, we examined the response of the mature tRNAome in infected hepatocytes to cocultured PBMCs, focusing on 46 distinct duck tRNA species. Dynamic profiling of the mature tRNAome indicated that 24 h of coculture significantly altered the mature tRNAome in DPHs (Additional file 6). This time point was selected to assess the influence of the quantity of PBMCs on tRNAome remodelling, as well as the effect of the infection dose of DHAV (Figures 3A, B). Our findings indicated that only a high concentration of PBMCs led to significant remodelling of the mature tRNAome in hepatocytes (Figure 3A). Notably, an increase in the number of cocultured PBMCs corresponded with a more pronounced decrease in the mature tRNAome. This observation implies that the degree of remodelling in the mature tRNAome is correlated with the quantity of cocultured PBMCs, particularly at levels sufficient to elicit increased transcription of proinflammatory cytokines (Additional file 5). Furthermore, the titres of DHAV were also linked to the remodelling of the mature tRNAome in DPHs, especially when 2.0 × 10^7^ copies/well of DHAV were inoculated (Figure 3B). This infection dose also significantly triggered increased cytokine transcription (Additional file 5). Notably, the tRNAome data are well aligned with findings from our recent proteomic studies. Using quantitative proteomic technology, we studied proteomic changes in duckling livers infected with DHAV at 24 hpi. Compared with those in the uninfected controls, we observed significant changes in numerous proteins related to tRNA biological processes, mostly tRNA-charging enzymes (ligases) (Figure 4). These enzymes are critical for catalysing the aminoacylation of tRNAs to produce aminoacyl-tRNAs [20]. In addition, DHAV infection has been shown to modulate host mRNA and miRNA profiles, as identified through miRNA sequencing [33]. Furthermore, reanalysis of these data revealed that DHAV infection increased the production of tRNA-derived stress-induced RNAs (tiRNAs) and tRNA-derived fragments (tRFs) (see Additional file 7), suggesting that the downregulation of the mature tRNAome may be associated with tRNA fragmentation.

### Indirect contact between PBMCs and infected DPHs mildly amplifies proinflammatory cytokine transcription and spares tRNAome remodelling

**Figure 5:**
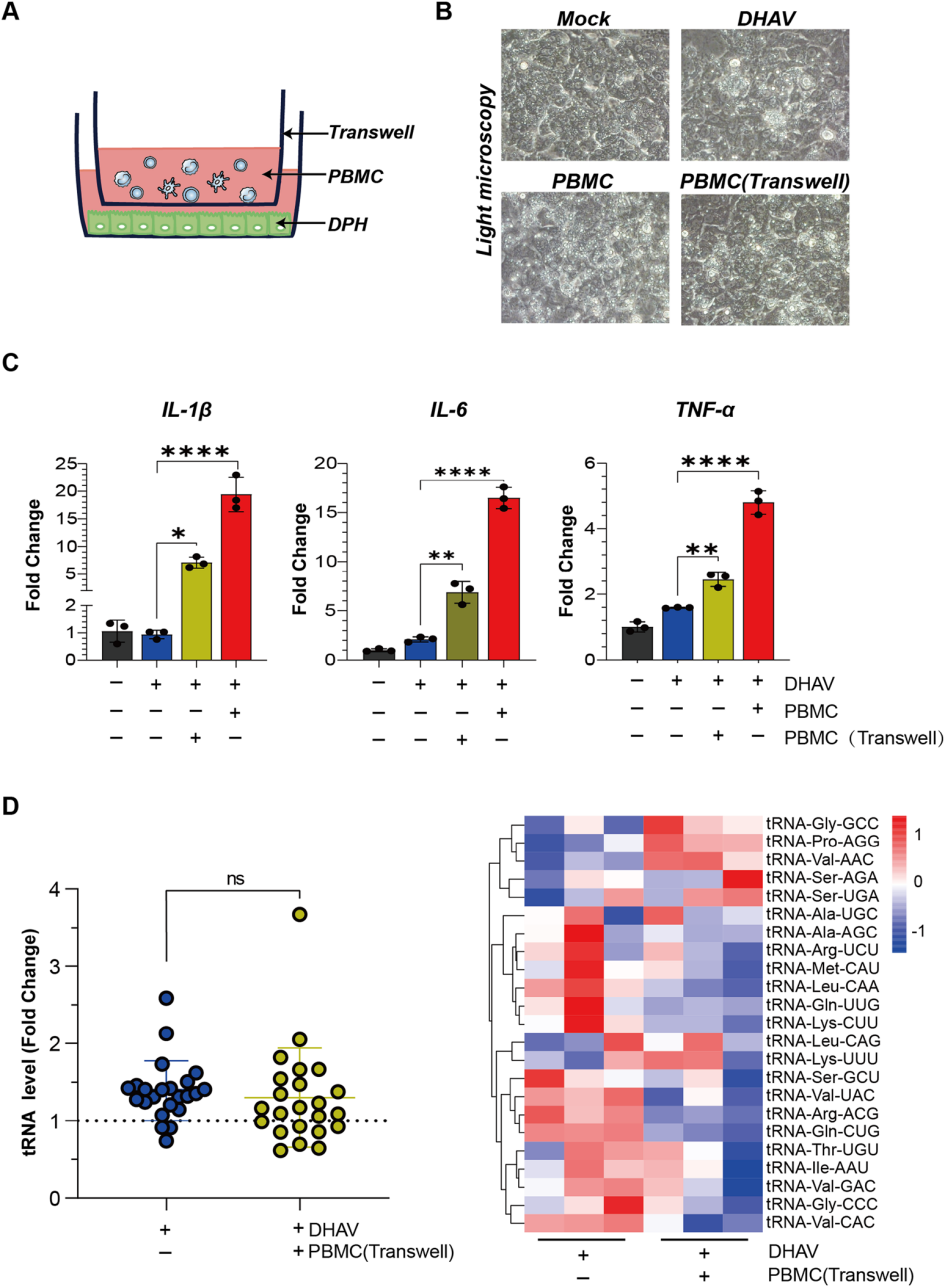
**The impact of indirect interactions between PBMCs and DHAV-infected DPHs on cytokine transcription and tRNAome remodelling**.** A** Schematic representation of the Transwell system cocultured with DHAV-infected DPHs and PBMCs. **B** Light microscopy images of DPHs under four different conditions: (i) Mock group: uninfected DPHs; (ii) DHAV group: DPHs infected with DHAV (2.0 × 10⁷ copies per well); (iii) PBMC group: coculture of DHAV-infected DPHs and PBMCs (5.0 × 10^6^ cells per well); (iv) PBMC (Transwell) group: coculture of DHAV-infected DPHs with PBMCs, in which the PBMCs were separated through a Transwell chamber. **C** Transcriptional quantification of IL-1β, IL-6, and TNF-α in infected DPHs when directly or indirectly cocultured with PBMCs. The expression levels of these genes were compared (*n* = 3). Statistical significance was determined via unpaired t tests; *, *p* < 0.05; **, *p* < 0.01; ***, *p* < 0.001. **D** Quantification of 23 selective tRNAs in DPHs cocultured with or without Transwell-separated PBMCs. The tRNAome data were normalized to those of the uninfected group (*n* = 3).

In liver biopsies from human patients with acute viral hepatitis, lymphocytes can physically interact with hepatocytes or communicate indirectly through their secreted cytokines [39]. Here, we investigated whether the indirect interaction of PBMCs with infected DPHs promotes proinflammatory cytokine responses. To this end, we used a Transwell coculture system in which PBMCs and DPHs were seeded separately in the inner and outer chambers (Figure 5A). Under a light microscope, no adhesion of PBMCs to DPHs was observed, indicating that only indirect interactions occurred (Figure 5B). We found that the separation of PBMCs via a Transwell system still upregulated proinflammatory cytokines within DPHs; however, the extent of upregulation was weaker than that in the direct coculture system (Figure 5C). Given that the remodelling of the tRNAome was subject to global alterations in the aforementioned assays (Figures 3A, B), the 23 tRNA species were selected as representatives of the mature tRNAome. In addition, the indirect interaction of PBMCs did not seem to remodel the hepatocyte tRNAome, which was not statistically significant (Figure 5D).

### Monocyte subpopulations in PBMCs orchestrate both proinflammatory cytokine transcription and tRNAome remodelling in infected DPHs

**Figure 6:**
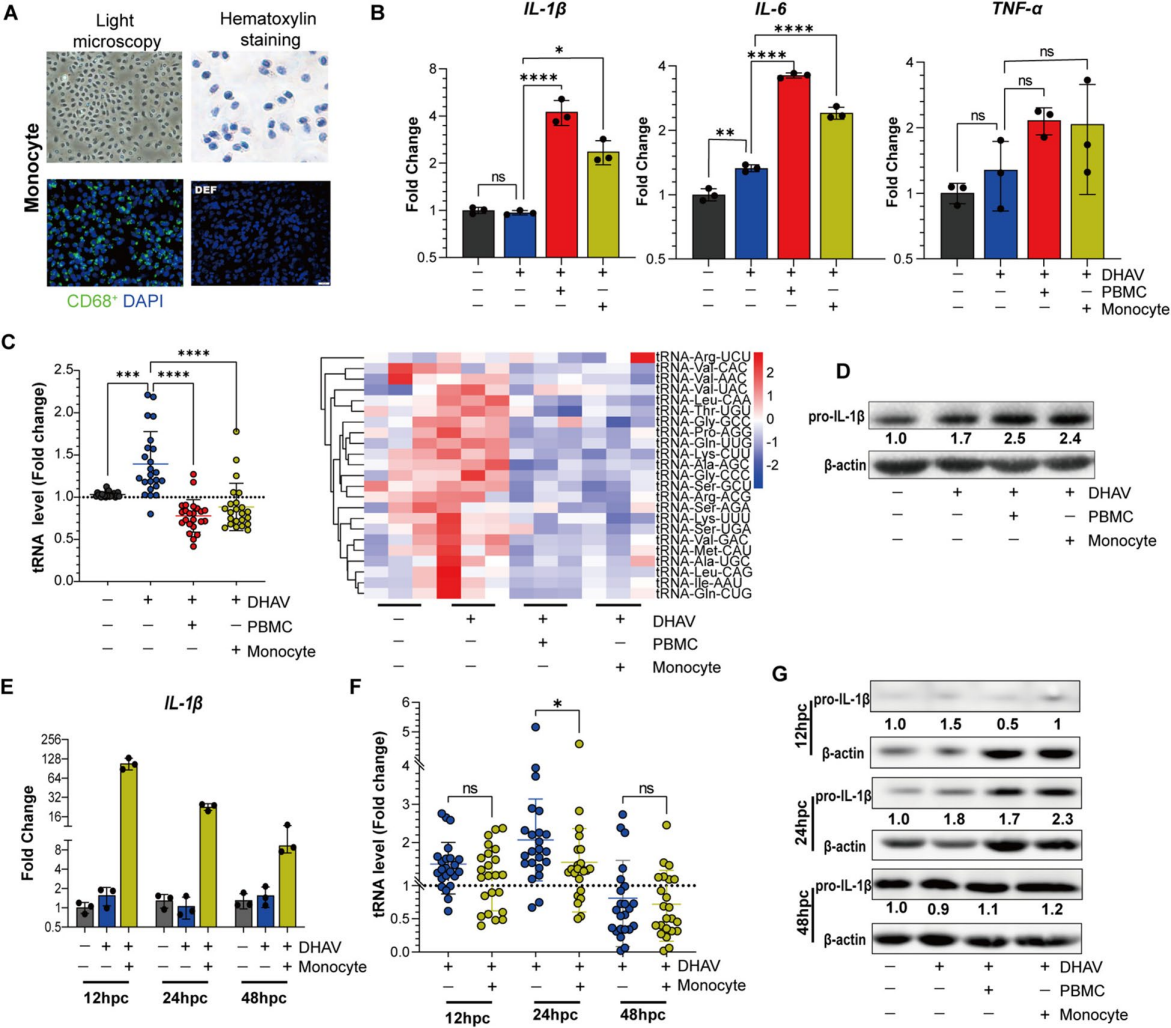
**Coordination of the hepatocyte IL-1β transcription-translation-mature tRNAome axis by monocytes**.** A** Representative images of monocytes (light microscopy and hematoxylin staining). The identification of monocytes was confirmed by a rabbit anti-CD68 polyclonal antibody (green), and the results were compared with those of DEFs. DAPI was used for nuclear staining. **B** The impact of monocytes on the transcription of proinflammatory cytokines in infected hepatocytes. The hepatocytes were cocultured with a total of 5.0 × 10^6^ PMBCs per well or with the corresponding monocytes derived from the same number of PBMCs. A total of 2.0 × 10^7^ copies per well of DHAV were subsequently used. Samples were collected at 24 hpc. RT‒qPCR was used to quantify the expression of IL-1β, IL-6, and TNF-α compared with that in uninfected DPHs (*n* = 3). Statistical significance was determined via unpaired t tests; *, *p* < 0.05; **, *p* < 0.01; ***, *p* < 0.001. **C** The impact of PBMCs or monocytes on the 23 selected tRNAs in infected DPHs. The tRNA data were normalized to those of the uninfected DPHs (*n* = 3). **D** Western blotting of the IL-1β protein in infected DPHs cocultured with PBMCs or monocytes at 24 hpc, with the same number of cells used as in panel B. The gray value of the IL-1β protein was then quantitatively evaluated via ImageJ software. **E** Dynamic changes in the expression of IL-1β mRNA in DHAV-infected DPHs cocultured with monocytes from 12 to 48 hpc (*n* = 3). DHAV-infected DPHs lacking monocyte coculture were used as a control, and uninfected DPHs were used as a negative control for gene normalization. **F** Dynamic changes in the 23 selected tRNAs in DPHs cocultured with monocytes. The tRNA data were normalized to those of the uninfected DPHs (*n* = 3). **G** Western blotting of IL-1β in infected DPHs during different durations of coculture with monocytes (12 hpc, 24 hpc, and 48 hpc). The amount of IL-1β protein was determined by analysing the gray value via ImageJ software.

In the previous approach, monocytes among PBMCs were easily isolated via the plate attachment method [30]. When examined, the monocytes presented several typical features, with misaligned nuclei that appeared polymorphic, such as oval, renal, and irregular nuclei (Figure 6A), which were also identified by a rabbit anti-CD68 polyclonal antibody [31]. Then, we specifically cocultured infected DPHs with these monocytes. We observed that monocytes were still able to trigger the transcription of proinflammatory cytokines (Figure 6B). Importantly, monocytes not only orchestrated the proinflammatory cytokine response but also remodelled the mature tRNAome in DPHs (Figure 6C). Both effects were similar to the effects observed in PBMCs. These data strongly suggest that monocytes constitute the main PBMC subpopulation that predominantly orchestrates both hepatocyte proinflammatory cytokine transcription and mature tRNA remodelling.

### Monocytes orchestrate the IL-1β-mediated hepatocyte proinflammatory response by coordinating IL-1β transcription and translation and mature tRNAome remodelling

Among all proinflammatory cytokines, IL-1β is a crucial mediator of liver inflammation and is commonly associated with liver inflammation [40]. At 24 hpc, monocytes promoted the protein translation of IL-1β, similar to the effect observed in PBMCs (Figure 6D). We further investigated the dynamic impacts of monocytes on both IL-1β and mature tRNAome remodelling from 12 to 48 hpc, as well as dynamic changes in IL-1β protein translation. As a result, we found that monocytes highly upregulated IL-1β transcription, with its mRNA level peaking at 12 hpc nearly 130-fold and gradually decreasing (Figure 6E). Similarly, we again observed significant remodelling of the mature tRNAome in the monocyte group at 24 hpc compared with that in the DHAV infection group, and the mature tRNAome was further downregulated at 48 hpc in both groups (Figure 6F).

Compared with those in the DHAV infection group, even when the mature tRNAome was downregulated at 24 hpc, the monocytes still promoted IL-1β protein synthesis (Figures 6F, G). At 48 hpc, when the mature tRNAome was further downregulated in both groups, the promotion of the IL-1β protein by the cocultured monocytes was largely reduced (2.3-fold versus 1.2-fold). However, increased IL-1β mRNA levels were detected (Figures 6F, G). These observations clearly indicate that monocytes cocultured with DPHs sustainably and dynamically modulate the transcription and translation of IL-1β and the remodelling of the mature tRNAome.

### Blocking the supernatant IL-1β protein inhibits the downstream transcription of IL-1β signalling and interferon genes

**Figure 7:**
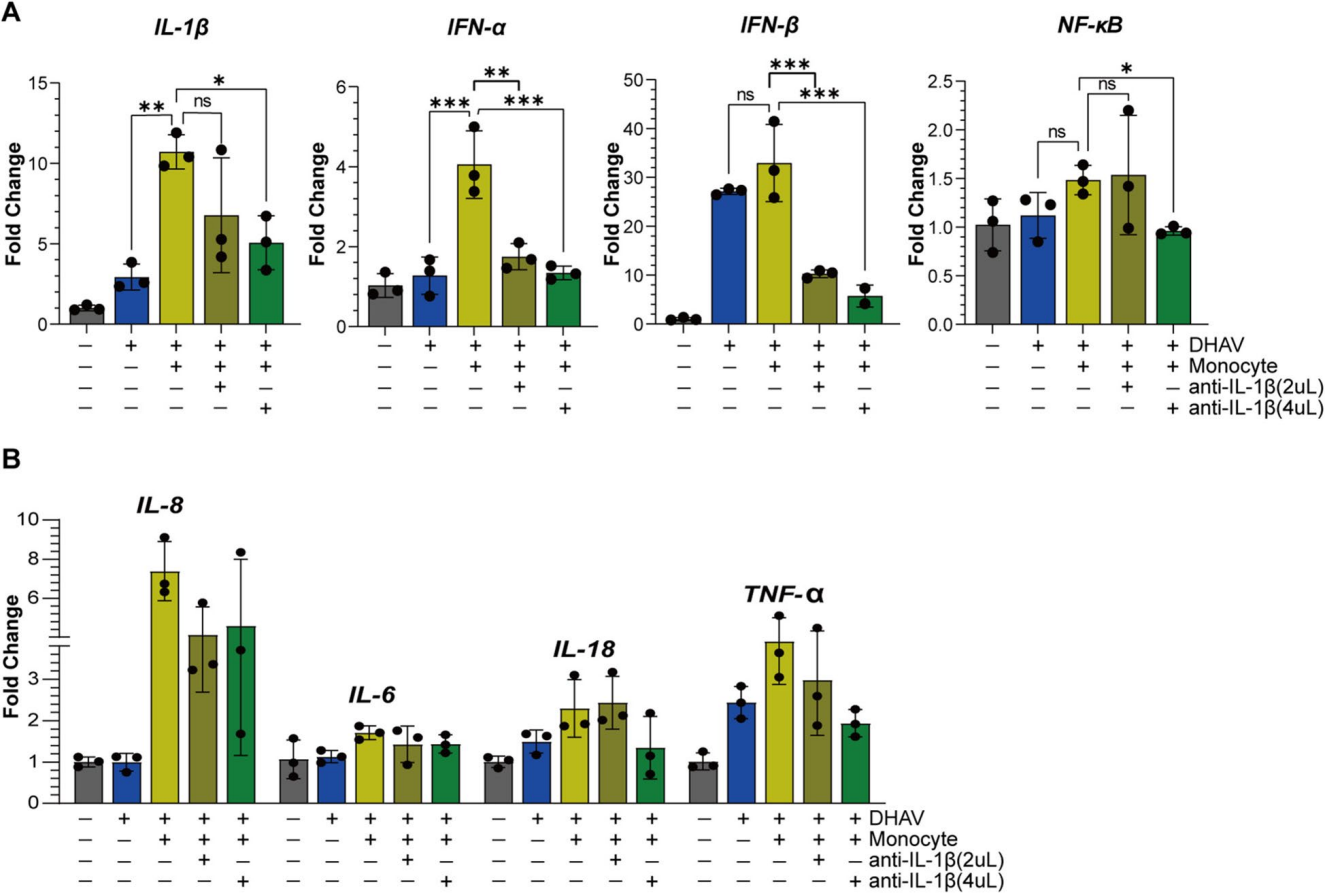
**Effects of neutralizing the IL-1β protein on downstream cytokine expression and interferon levels**. **A** Monocytes were preincubated with 2 µL and 4 µL of rabbit anti-IL-1β antibody before being added to the DPH and monocyte coculture system. qPCR was used to quantify the levels of IL-1β, IFN-α, IFN-β, and NF-κB. Three biological replicates were employed. Statistical significance was determined via unpaired t tests; *, *p* < 0.05; **, *p* < 0.01; ***, *p* < 0.001. **B** qPCR quantification of IL-8, IL-6, IL-18, and TNF-α levels (*n* = 3).

Since IL-1β plays a significant role in liver inflammation [15], blocking its proinflammatory effects is possible. The blockade of IL-1α or IL-1β by interleukin-1 receptor antagonists or antibodies has been consistently demonstrated to be a practical approach for treating IL-1-related diseases, such as T-cell-induced cytokine release syndrome [36]. In this study, we exploited duck IL-1β-specific antibodies to evaluate the therapeutic potential of blocking IL-1β in our coculture model. To ascertain the impact of the rabbit anti-duck IL-1β antibodies, 2 mg/mL and 4 mg/mL antibodies were utilized, as were potential dose-dependent effects. After 24 h of IL-1β blockade [34], the transcription levels of IL-1β downstream cytokines, such as IL-1β, IL-6, TNF-α, IL-8, IFN-β, and NF-κB, were quantified [41], as were the levels of the innate antiviral IFN-α and IL-18, which are members of the IL-1 family. We found that blocking the IL-1β protein significantly reduced its self-transcription and NF-κB transcription (Figure 7A). NF-κB regulates immune responses and the inflammatory process by promoting the transcription of multiple proinflammatory cytokines [42]. However, other cytokines, such as IL-6, IL-8, IL-18, and TNF-α, were minimally reduced (Figure 7B). To our surprise, several unconventional downstream cytokines of IL-1β signalling, including IFN-α and IFN-β, were significantly downregulated (Figure 7A). These findings suggest that monocytes may play a role in the antiviral response. In support of these findings, our data revealed an antiviral effect exerted by monocytes that was relatively low (maximum of 50%) in comparison with their impact on the proinflammatory response (Additional file 8). Overall, we revealed that monocytes triggered a hepatocyte inflammatory response through transcriptional activation of proinflammatory cytokines and tRNAome remodelling-mediated translation coordination.

## Discussion

Owing to the importance of liver-infiltrated lymphocytes, a consistent impetus exists to study their role in hepatitis pathogenesis, such as Kupffer cells and T cells. In an HAV infection mouse model, virus-specific CD8 + T cells were associated with liver inflammation and likely mediated acute hepatitis A [7]. In comparison, other mouse model studies have suggested that Kupffer cells drive liver inflammation by activating proinflammatory signalling pathways and the transcription of corresponding proinflammatory cytokines [9]. We recently showed that infection of monocytes with HEV, a type of progenitor cell of Kupffer cells, promoted IL-1β-mediated inflammasome activation [15]. The existing evidence suggests that liver-resident immune cells contribute to liver inflammation, but it is not known whether their contact with hepatocytes contributes to liver inflammation. Using a PBMC and hepatocyte coculture system, we further revealed an unrecognized role of PBMCs in promoting the hepatocyte inflammatory response, especially the monocyte subpopulation that drives both proinflammatory cytokine transcription and the mature tRNAome. As a proof-of-concept, we demonstrated that IL-1β protein synthesis, its mRNA transcription and mature tRNAome remodelling are coordinately and dominantly modulated by cocultured monocytes.

We found that coculture of PBMCs robustly amplified the transcription of proinflammatory cytokines, particularly IL-1β (~130-fold), in hepatocytes (Additional file 5), in which monocytes are predominantly effector cells (Figure 6A). However, little is known about the mechanisms by which monocytes drive high proinflammatory cytokine transcription in hepatocytes. One potential mechanism could be chromatin remodelling, which drives specifically proinflammatory cytokine transcription [43]. Additionally, sustained activation of RNA polymerase II, which mediates consistent cytokine transcription, may play a role [44]. In addition, the modulation of transcription factors such as phosphorylation can increase transcription activity [42]. Therefore, we believe that monocytes may drive the hepatocyte transcription machinery or accessible regulatory elements for high cytokine expression; this remains an interesting question to be answered in the future.

As a critical mediator of inflammation, IL-1β plays a pivotal role in inflammasome activation-mediated inflammation [15]. Upon the activation of pattern recognition receptors (PRRs) or danger-associated molecular patterns (DAMPs), a signalling cascade is initiated, leading to the synthesis of pro-IL-1β [41]. The inactive form of pro-IL-1β is then cleaved to form active or secreted IL-1β. Secreted IL-1β further activates downstream signalling cascades through the NF-κB and MAPK pathways, ultimately promoting the transcription of proinflammatory genes, thereby orchestrating the inflammatory response [42]. Indeed, we found that DPHs infected with DHAV triggered NF-κB and NLRP3 inflammasome-mediated IL-1β production via the viral 2B protein to disrupt the cellular homeostasis of Ca^2+^, K^+^, reactive oxygen species (ROS) and cathepsin [45]. Therefore, IL-1β blockade-based anti-inflammatory therapy has often been used to treat CRS and the side effects of cancer immunotherapy [34, 36]. Although we do not know whether monocytes further trigger IL-1β-mediated inflammasome activation, duck hepatocytes infected with only DHAV can also trigger IL-1β-mediated inflammasome activation [45], but the extent of IL-1β transcription is much lower than that in infected DPH cocultured with PBMCs (Figure 2C) or the monocyte subpopulation (Figure 6E). Despite the importance of the intermediate pathway that produces IL-1β, we found that monocytes promote IL-1β protein synthesis and that blocking IL-1β in the supernatant affects downstream signalling (Figures 6D and 7A). Another implication of this finding is that monocytes could be promising targets for immune therapy, as this cell population robustly drives the production of the IL-1β protein in hepatocytes.

The mature tRNAome performs translational decoding of virtually all transcripts within cells. Thus, the modulation of the mature tRNAome affects gene translational control, especially for highly transcribed cytokines such as IL-1β (Figure 2C). Our findings revealed that the mature tRNAome in monocyte-cultured hepatocytes was sequentially downregulated at 24 hpc and 48 hpc (Figures 3A, B and 6F). Interference at any step in tRNA biosynthesis could lead to tRNA remodelling: (a) interference with RNA Pol III activity, which mediates tRNA gene transcription [46, 47]; (b) transcriptional interference with RNA-Pol II, which mediates the transcription of protein-encoding genes. RNA-Pol II and III are biologically connected and compete when coregulation occurs [48]; the former process synthesizes mRNAs, whereas the latter produces a tRNAome that performs translational decoding of these mRNAs. Coherently, we detected high transcription of proinflammatory cytokines such as IL-1β (Figure 6B), accompanied by simultaneous downregulation of the mature tRNAome (Figure 6C), which is consistent with our recent observations [15].

Additionally, tRNA fragmentation should play a role in tRNAome downregulation. In the DHAV-infected DEFs, some tRNA-derived fragments, such as tRFs and tiRNAs, were dramatically increased in comparison with those in the uninfected group (Additional file 7). Although the data are not derived from cocultured hepatocytes, this finding indicates the plausibility of tRNA fragmentation-mediated tRNAome downregulation. To cope with tRNAome downregulation, reducing the activity of tRNA-aminoacyl enzymes would be beneficial because their substrates, tRNAs, are reduced [20]. Coherently, we found that the protein levels of many tRNA-charging enzymes (ligases) were significantly decreased in the livers of DHAV-infected ducklings (Figure 4).

Our study revealed that monocytes play a key role in hepatocyte inflammatory responses. This occurred through a mechanism that orchestrated the transcription of proinflammatory cytokines and remodelling of the mature tRNAome. We provided evidence demonstrating the functional coordination of IL-1β transcription, the mature tRNAome, and protein synthesis. In addition to the well-known role of monocytes in liver inflammation, we further illustrated a paradigm in which these monocytes promote inflammatory responses in the hepatocytes with which they interact. We envision that the findings of this study will provide new insights into the complicated role of cell-to-cell interplay in liver inflammation, specifically the interaction between monocytes and hepatocytes.

## Supplementary Information

**Additional file 1. Primers used for measuring duck cytokines and DHAV.**

**Additional file 2. Primers used for the quantification of the mature duck tRNAome.**

**Additional file 3. Morphological and structural changes in fibroblast-like hepatocytes.** The images illustrate the morphological and structural alterations that occur before and after fibroblast-like hepatocyte transformation. The images were obtained via light microscopy, with samples stained with Oil Red O, Masson’s trichrome, and DAPI.

**Additional file 4. Evaluation of hepatocyte RNA contamination from adherent monocytes in the coculture system.** The contamination of hepatocyte RNA can be attributed exclusively to adherent monocytes, with a range of 7% to 11% across three independent replicates and three biological replicates. The other suspended cells from the PBMCs can be easily eliminated through appropriate washing during sample collection. The RNA content was normalized to that of the hepatocytes that were not cocultured (which was set as 1.0).

**Additional file 5. Effects of the number of PBMCs, the DHAV titre, and the coculture time on the proinflammatory cytokine response in infected DPHs.** qPCR was used to quantify the IL-1β, IL-6, and TNF-α levels in cocultures with varying numbers of PBMCs. The number of PBMCs in the cocultures was varied: 5.0 × 10^6^, 5.0 × 10^5^, and 5.0 × 10^4^ per well, with three biological replicates for each group. We used 2.0 × 10^7^ copies per well of DHAV, with RNA samples collected at 24 hpc. **B** qPCR quantification of IL-1β, IL-6, and TNF-α levels in response to inoculation with different doses of DHAV, such as 2.0 × 10^7^, 2.0 × 10^6^, and 2.0 × 10^5^ copies per well. A total of 5.0 × 10^6^ PBMCs per well were used, and RNA samples were collected at 24 hpc. C qPCR was used to quantify the levels of IL-1β, IL-6, and TNF-α at different times of coculture: 6 hpc, 12 hpc, 24 hpc, and 48 hpc (*n* = 3). In each well, a total of 2.0 × 10^7^ copies of DHAV and 5.0 × 10^6^ PBMCs were used, with RNA samples collected at the indicated time points.

**Additional file 6. Dynamic profiling of the mature tRNAome.** Twenty-three selected tRNAs were profiled at different times of coculturing: 6 hpc, 12 hpc, 24 hpc and 48 hpc (*n* = 3). A total of 2.0 × 10^7^ copies per well of DHAV and 5.0 × 10^6^ PBMCs per well were used. The tRNA data were normalized to those of the DPHs after 6 h of DHAV infection.

**Additional file 7. DHAV infection is associated with tRNA fragmentation.** Differential expression of tiRNAs and tRFs in DHAV-infected vs. uninfected fibroblasts (*n* = 3).

**Additional file 8. The impact of monocytes on DHAV replication in cocultured DPHs.** qPCR quantification of DHAV RNA in DPHs cocultured with or without monocytes at 24 hpc (*n* = 3).

## Data Availability

The data used and/or analysed are available in this paper or in a public dataset disposition platform.
