# Supplementary figures and images for: Orchestration of hepatocyte inflammatory responses by monocytes during acute viral hepatitis in ducks in vitro
Source: Vet Res. 2025 Oct 17;56:200. doi: 10.1186/s13567-025-01630-9 (PMC12534983; doi:10.1186/s13567-025-01630-9)

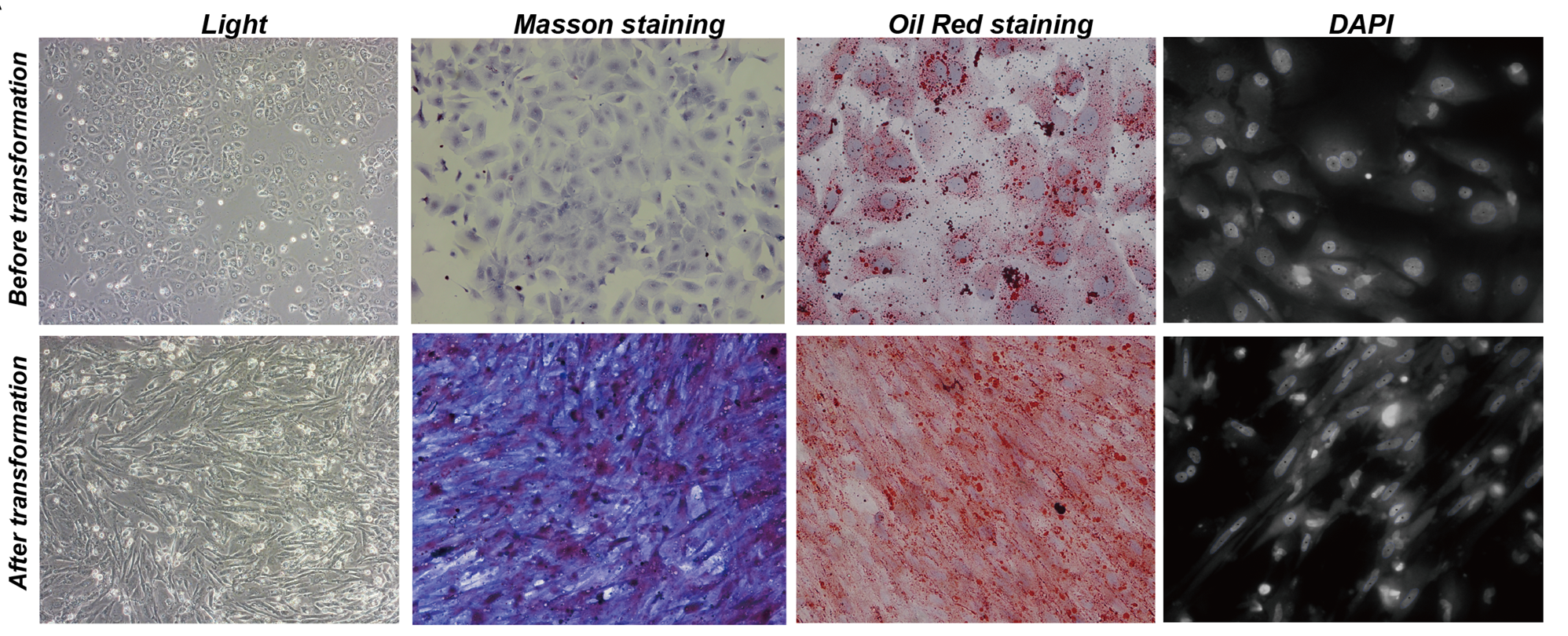

Supplement: Supplementary file 3 — Additional file 3. Morphological and structural changes in fibroblast-like hepatocytes. The images illustrate the morphological and structural alterations that occur before and after fibroblast-like hepatocyte transformation. The images were obtained via light microscopy, with samples stained with Oil Red O, Masson’s trichrome, and DAPI. [file 13567_2025_1630_MOESM3_ESM.png]

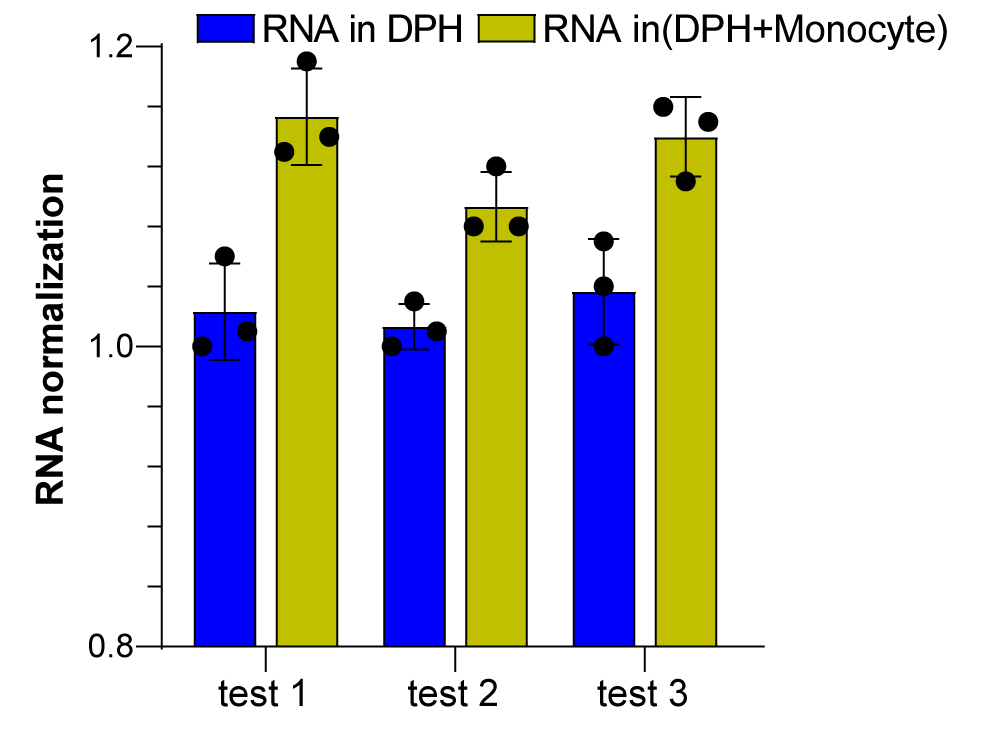

Supplement: Supplementary file 4 — Additional file 4. Evaluation of hepatocyte RNA contamination from adherent monocytes in the coculture system. The contamination of hepatocyte RNA can be attributed exclusively to adherent monocytes, with a range of 7% to 11% across three independent replicates and three biological replicates. The other suspended cells from the PBMCs can be easily eliminated through appropriate washing during sample collection. The RNA content was normalized to that of the hepatocytes that were not cocultured (which was set as 1.0). [file 13567_2025_1630_MOESM4_ESM.tiff]

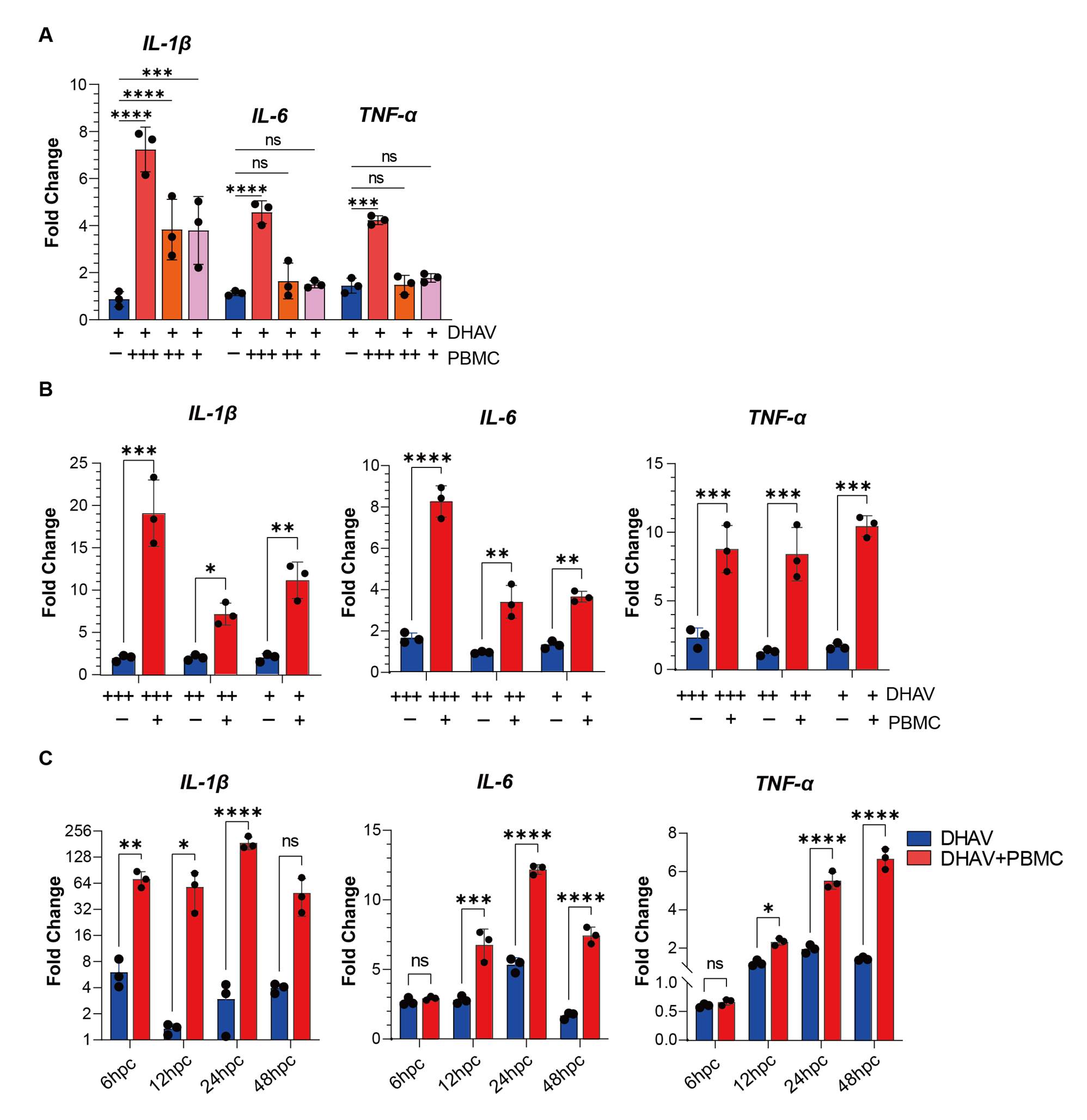

Supplement: Supplementary file 5 — Additional file 5. Effects of the number of PBMCs, the DHAV titre, and the coculture time on the proinflammatory cytokine response in infected DPHs. qPCR was used to quantify the IL-1β, IL-6, and TNF-α levels in cocultures with varying numbers of PBMCs. The number of PBMCs in the cocultures was varied: 5.0 × 10^6^, 5.0 × 10^5^, and 5.0 × 10^4^ per well, with three biological replicates for each group. We used 2.0 × 10^7^ copies per well of DHAV, with RNA samples collected at 24 hpc. B qPCR quantification of IL-1β, IL-6, and TNF-α levels in response to inoculation with different doses of DHAV, such as 2.0 × 10^7^, 2.0 × 10^6^, and 2.0 × 10^5^ copies per well. A total of 5.0 × 10^6^ PBMCs per well were used, and RNA samples were collected at 24 hpc. C qPCR was used to quantify the levels of IL-1β, IL-6, and TNF-α at different times of coculture: 6 hpc, 12 hpc, 24 hpc, and 48 hpc (n = 3). In each well, a total of 2.0 × 10^7^ copies of DHAV and 5.0 × 10^6^ PBMCs were used, with RNA samples collected at the indicated time points. [file 13567_2025_1630_MOESM5_ESM.png]

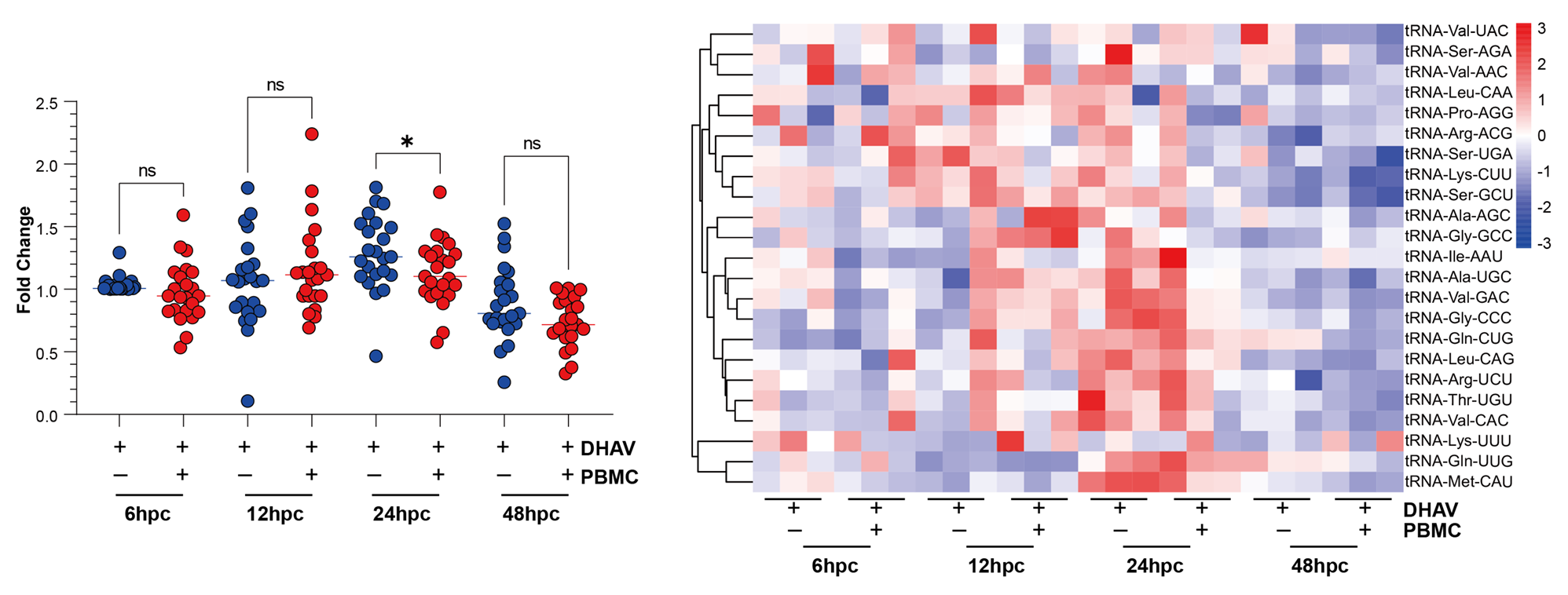

Supplement: Supplementary file 6 — Additional file 6. Dynamic profiling of the mature tRNAome. Twenty-three selected tRNAs were profiled at different times of coculturing: 6 hpc, 12 hpc, 24 hpc and 48 hpc (n = 3). A total of 2.0 × 10^7^ copies per well of DHAV and 5.0 × 10^6^ PBMCs per well were used. The tRNA data were normalized to those of the DPHs after 6 h of DHAV infection. [file 13567_2025_1630_MOESM6_ESM.png]

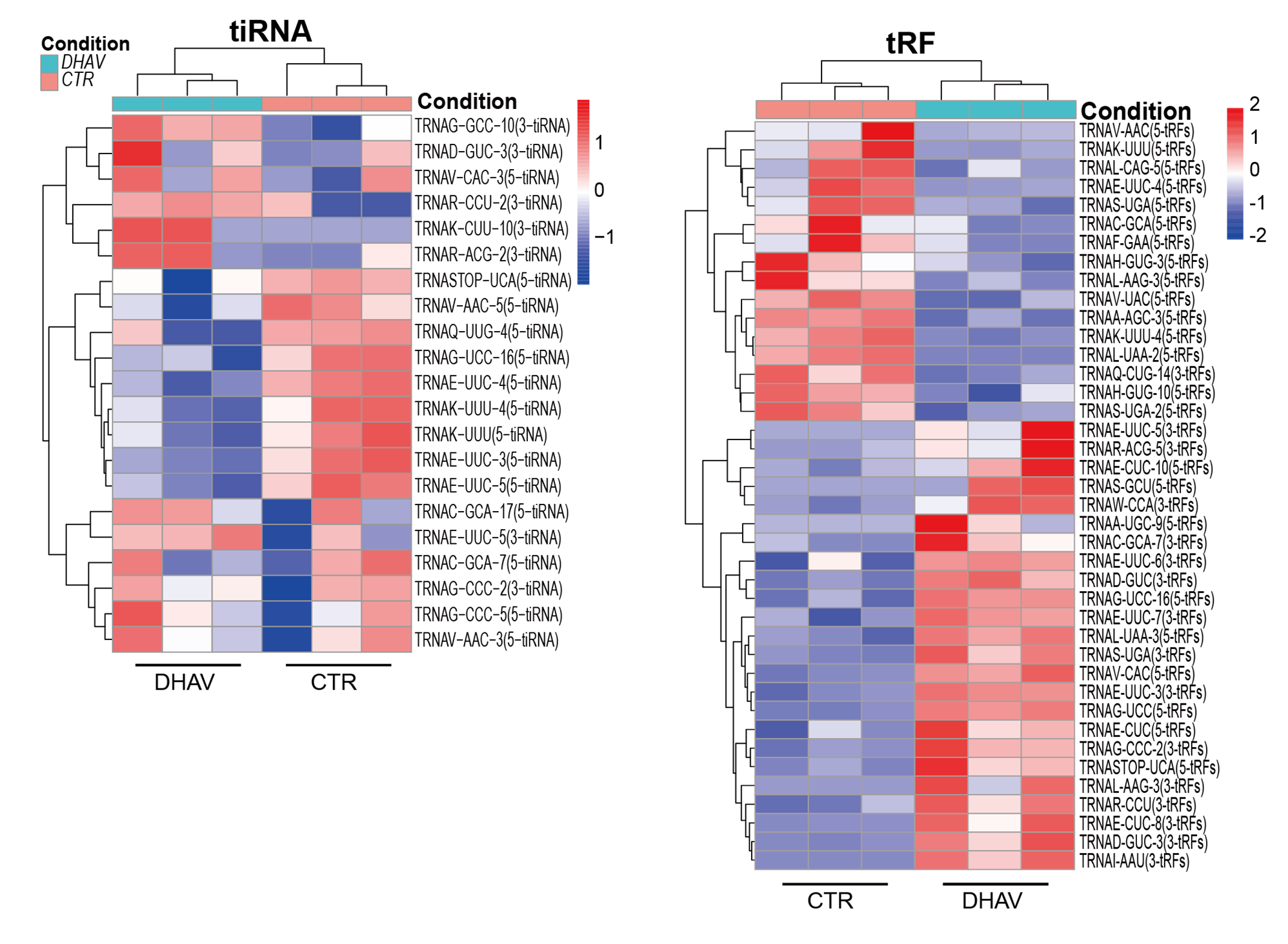

Supplement: Supplementary file 7 — Additional file 7. DHAV infection is associated with tRNA fragmentation. Differential expression of tiRNAs and tRFs in DHAV-infected vs. uninfected fibroblasts (n = 3). [file 13567_2025_1630_MOESM7_ESM.png]

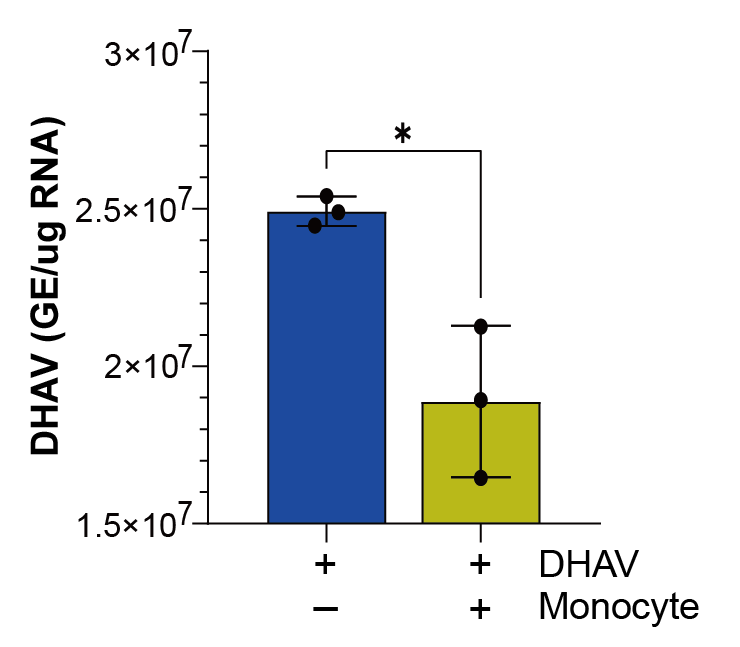

Supplement: Supplementary file 8 — Additional file 8. The impact of monocytes on DHAV replication in cocultured DPHs. qPCR quantification of DHAV RNA in DPHs cocultured with or without monocytes at 24 hpc (n = 3). [file 13567_2025_1630_MOESM8_ESM.png]
